# Inhibitory Effects of Six Types of Tea on Aging and High-Fat Diet-Related Amyloid Formation Activities

**DOI:** 10.3390/antiox10101513

**Published:** 2021-09-24

**Authors:** Juan Wan, Meiyan Feng, Wenjing Pan, Xin Zheng, Xinya Xie, Baozhu Hu, Cuiqin Teng, Yingzi Wang, Zhonghua Liu, Jianhua Wu, Shuxian Cai

**Affiliations:** 1 National Research Center of Engineering Technology for Utilization of Botanical Functional Ingredients, Hunan Agricultural University, Changsha 410128, China; wanjuan@stu.hunau.edu.cn (J.W.); fengmeiyan@stu.hunau.edu.cn (M.F.); pwj0204@stu.hunau.edu.cn (W.P.); zhengxin@stu.hunau.edu.cn (X.Z.); xxy999@stu.hunau.edu.cn (X.X.); HU0707@stu.hunau.edu.cn (B.H.); wyz19860908@163.com (Y.W.); 2 Key Laboratory of Ministry of Education for Tea Science, Hunan Agricultural University, Changsha 410128, China; 3 Co-Innovation Center of Education Ministry for Utilization of Botanical Functional Ingredients, Hunan Agricultural University, Changsha 410128, China; 4 Wuzhou Institute of Agricultural, Wuzhou 543003, China; teng3000@163.com (C.T.); wjh0056@163.com (J.W.)

**Keywords:** six types of tea, amyloid, senescence, high-fat diet, antioxidant capacity, catechins

## Abstract

Aging and lipid metabolism disorders promote the formation and accumulation of amyloid with β-sheet structure, closely related to cardiovascular disease, senile dementia, type 2 diabetes, and other senile degenerative diseases. In this study, five representative teas were selected from each of the six types of tea, and a total of 30 teas were selected to evaluate the inhibitory activities on the formation of aging-related amyloid in vitro. The results showed that the 30 teas had a significant inhibitory effect on the formation activity on aging-related amyloid at the protein level in vitro. Although the content of catechins is relatively low, black tea and dark tea still have significant antioxidant activity and inhibit the formation of amyloid. A high-fat diet established the model of lipid metabolism disorder in premature aging SAMP8 mice, and these mice were gavaged different tea water extracts. The results showed that different tea types have a significant inhibitory effect on the formation of β-amyloid and Aβ42 mediated by age-related lipid metabolism disorders, and the in vivo activity of fully fermented teas was better than that of green tea. The action mechanism was related to antioxidation, anti-inflammatory, and improving lipid metabolism.

## 1. Introduction

Amyloid is self-assembled by different proteins into fibrous aggregates with similar morphology and rich in β-sheet structure [1,2,3]. The β-sheet structure is the seed of aggregate formation [4]. Fibrils rich in β-sheet structure can interact with the cell membrane, destroy the phospholipid bilayer of the cell membrane, increase membrane permeability, and cause cell apoptosis [5,6]. Modern studies have identified that about 60 kinds of proteins can form fibrous aggregates, and at least 36 are associated with human amyloid diseases such as cardiovascular disease, Alzheimer’s disease, type 2 diabetes, and other senile degenerative diseases [7,8,9,10,11]. Under stress conditions such as oxidative stress, inflammation, and metabolic disorders, biomacromolecules such as protein, nucleic acid, and fat tend to aggregate [12]. Therefore, timely and effective inhibition of the formation of proteins rich in β-sheet structure plays a vital role in inhibiting amyloid and preventing degenerative diseases.

Obesity, metabolic disorders, and oxidative stress induced by aging and high-fat diets can promote the accumulation of amyloid and the incidence rate of Alzheimer’s disease [13,14,15,16,17,18,19]. Toxic carbonyl compounds with conjugated and electron-deficient structures produced by aging and lipid peroxidation can be continuously linked with Cys, His, Lys, and other nucleophilic amino acid residues of biological macromolecules [20,21]. In addition, these toxic carbonyl compounds can also bind with the proteasome and destroy the cell proteolysis system, and further lead to the deposition of β-sheet-rich protein aggregates [22]. At present, statins and antibodies have shown sound therapeutic effects in clinical practice [23,24], but their side effects cannot be ignored.

Tea and its functional components inhibit the production of amyloid. Clinical studies have shown that drinking tea can alleviated the cognitive impairment caused by amyloid [25,26,27,28,29]. Cell and animal experiments have proved that tea water extract can inhibit the production of amyloid. Tea polyphenols, especially epigallocatechin gallate (EGCG), can significantly inhibit the formation of β-sheet structure [30,31,32]. EGCG and theaflavins can inhibit the carbonyl-amino cross-links reaction and inhibit the aging-related amyloid [33]. A kind of flavoalkaloids condensed between catechins and theanine is found in aged white tea and post-fermented dark tea, and its antioxidant activity is better than that of catechins. In the senescent SAMP8 mice model, these flavoalkaloids can downregulate the pathway of amyloid formation and significantly inhibit the formation of amyloid in the brain [34].

Tea has antioxidation, anti-inflammation, anti-tumor, anti-atherosclerosis, and delaying neurodegenerative diseases and other health functions [35]. Chinese tea is mainly divided into six types: green tea, white tea, yellow tea, oolong tea, black tea, and dark tea. Green tea belongs to unfermented tea, and the content of tea polyphenols is approximately 20–35%, of which EGCG accounts for 50–60% of tea polyphenols [36]. Both white tea and yellow tea are lightly fermented teas, and their active ingredients are similar to green tea. Oolong tea is semi-fermented tea made from mature fresh leaves and is richer in tea polyphenols. Black tea is a fully-fermented tea. During its processing, tea polyphenols undergo oxidation, polymerization, condensation, and other reactions to further form theaflavins, thearubigins and theabrownines, the contents of which are approximately 0.4–2%, 5–11%, and 4–8%, respectively. Dark tea is a post-fermented tea. Under the transformation of microorganisms, dark tea contains more organic acids, theabrownines and flavoalkaloids [37]. Among them, the content of tea polyphenols is approximately 5–10%, and the content of theabrownines is approximately 8–14%.

At present, research on tea’s inhibitory activity against amyloid mainly focuses on green tea and monomer components such as EGCG and theaflavins [38,39]. There are few studies on evaluating the inhibitory activity of six types of tea on amyloid formation. In this study, five representative teas were selected from each of the six types of tea, and a total of 30 teas were selected to evaluate the in vitro activity of different teas in inhibiting the formation of aging-related amyloid. On this basis, we further analyzed teas’ inhibitory activity and mechanism with different fermentation levels on the formation of amyloid caused by aging and high-fat diet. We provided a theoretical basis for how to drink tea to inhibit the formation of amyloid.

## 2. Materials and Methods

### 2.1. Materials

Thirty teas were purchased from China (for details, see Table 1). 1,1,3,3-Tetranethoxypropane (TMP) (98%) was purchased from Fluka (Seelze, Germany). Bovine serum albumin (BSA) (≥99%) and thioflavin T (ThT) were purchased from Sigma (Saint louis USA). Triglyceride (TG), total cholesterol (TC), superoxide dismutase (SOD), catalase (CAT), glutathione peroxidase (GSH-Px), malondialdehyde (MDA), and total antioxidant capacity (T-AOC) detection kits were purchased from Nanjing Jiancheng Biotechnology Co., Ltd., Nanjing, China. Adenosine triphosphate (ATP) Assay Kit was purchased from Beyotime Biotechnology Co., Ltd. (Shanghai, China). Mouse interleukin-6 (IL-6) and amyloid beta 42 (Aβ42) ELISA kits were purchased from Feiya Biotechnology Co., Ltd. (Haian, China). In addition to anti-GAPDH (Cell Signaling, Boston, MA, USA), the following primary antibodies were used for Western blotting analysis: anti-β-amyloid (Cell Signaling, Boston, MA, USA), anti-multiubiquitin (Medical & Biological Laboratories co. Ltd., Tokyo, Japan), anti-4-HNE (Millipore, Boston, USA), anti-sequestosome-1 (p62) (Epitomics, Burlingame, CA, USA), and anti-RAGE (Santa Cruz Biotechnology, Dallas, TX, USA). Western chemiluminescent horseradish peroxidase substrate was purchased from Millipore. Other reagents used in the experiment are of analytical grade or chromatographic purity grade.

### 2.2. Ethical Statement

All animals were kept in a specific pathogen-free facility at Peking University Health Science Center, and the experimental procedures and protocols were approved by the Institutional Animal Care and Use Committee of Peking University (NO SYXK (jing) 2016-0041). Animal experiments were conducted following the guidelines of the national animal care legislation. All operations were performed under pentobarbital sodium anesthesia to minimize the pain of experimental animals.

### 2.3. Preparation of 10 mol/L MDA

Malonaldehyde (MDA) was produced by acid hydrolysis of TMP [40]. In short, we added 4.5 mL of 1 mmol/L HCl to a 50 mL volumetric flask, accurately pipetted and added 0.0845 mL TMP, and put the volumetric flask in a 40 °C water bath while shaking and heating for about 2.5 min until the solution was uniform and clear. Then, we added about 1 mL of 6 mol/L NaOH to adjust the pH value of the solution to neutral. Finally, the solution was diluted to 50 mL with 0.1 mmol/L PBS (pH 7.0) and stored at 4 °C for later use.

### 2.4. Aging-Related Amyloid Preparation and Tea Processing

Under the condition of adding tea extract in the concentration range of 0 to 4000 μg/mL, BSA (1 mg/mL), and MDA (2 mmol/L) were incubated in PBS (pH 7.0) at 37 °C in a constant temperature blast drying oven for 24 h (added tea extract and MDA at the same time). The blank control group was added with an equal volume of PBS with the corresponding pH value [20]. The protein samples of different treatment groups were processed as follows [41]: added 1/5 of the total volume of the reaction system to 20% trichloroacetic acid (TCA) in the BSA/MDA reaction system, mixed well, centrifuged at 11,000× *g* for 3 min, and removed the supernatant; then we added 0.5 mL PBS and 0.1 mL 20% TCA, mixed well, centrifuged at 11,000× *g* for 3 min, removed the supernatant to ensure that the impurities in the samples were removed entirely. Finally, the precipitate was dissolved in 0.5 mL PBS to obtain protein samples of the treatment groups.

### 2.5. Fluorescence Intensity Detection and Analysis

The proteins of different treatment groups prepared in Section 2.4 were, respectively, dissolved in 0.5 mL PBS and diluted 10 times for detection. The fluorescence detection of the characteristic structure of aging-related amyloid was as follows: we pipetted 0.1 mL of the protein solution of different treatment groups into a 96-well plate and placed it on a multi-function microplate instrument (Varioskan Flash, Thermo, Waltham, MA, USA). The fluorescence intensity at 460 nm was detected under the excitation of 395 nm wavelength.

ThT was used to detect the content of amyloid β-sheet toxic seed structure. The detection method of ThT was as follows: pipetted 40 μL of protein solution of different treatment groups into a 96-well plate, and added 160 μL of ThT working solution to each sample well. Placed the 96-well plate on the multi-function microplate instrument and detected the fluorescence intensity with the emission wavelength of 485 nm under the excitation of 440 nm wavelength.

### 2.6. Determination of Antioxidant Capacity

Used T-AOC kit (Nanjing Jiancheng Biotechnology Co., Ltd., Nanjing, China) to test the antioxidant capacity of tea extracts, and operated strictly following the kit instructions. Accurately weighed 5 mg of tea extract and dissolved it in 10 mL of pure water, and diluted to 100, 200, 300, 400, and 500 µg/mL, respectively, then added ABTS working solution, reacted at room temperature for 6 min, and put it in the multi-functional microplate instrument to detect the absorbance at a wavelength of 406 nm.

### 2.7. Preparation and Determination of Physicochemical Components of Tea Extract

Accurately weighed 100 g of tea into a 2 L Erlenmeyer flask, poured boiling water, and the ratio of tea to water was 1:10. Filtered and extracted twice. The combined tea soup was cooled to room temperature, put into −20 °C pre-frozen, and then freeze-dried in a vacuum freeze dryer (Alpha 1-4/LSC Plus, Christ, Osterode, Germany) at −42 °C for 24 h to obtain the tea extract, and finally stored at −20 °C for use.

We accurately weighed 0.1 g of tea extract, dissolved it in 5 mL volumetric flask for ultrasonic dissolution, and passed the membrane. The HPLC method used for determining the contents of catechins and alkaloids was as follows: using a C18 chromatographic column, flowing A: pure water, B: N, N-dimethylformamide: methanol: glacial acetic acid = 39.5:2:1.5. The wavelength was 278 nm and the temperature was 30 °C, the flow rate of 1.0 mL/min, and the elution gradient was set as follows: 9% B (0.01 min), 14% B (10 min), 23% B (15 min), 36% B (27 min), 36% B (31 min), 9% B (32 min).

### 2.8. Animal Experiments

Select SPF-grade 8-week-old male normal control group with 10 SAM-resistant-1 (SAMR1) mice and 60 senescence-accelerated SAM-prone-8 (SAMP8) mice in an SPF-grade animal room for one week. Then, the SAMP8 mice were randomly divided into 7 groups according to body weight and were reared in a clean animal room for one week, with free food and water. Subsequently, they were randomly divided into 6 groups according to their weight. Both control groups R1 + ND and P8 + ND were given ordinary breeding feed, and the P8 + HFD and tea treatment groups were given a 60% high-fat diet for 4 weeks. After that, the tea treatment groups were intragastrically administered with 200 mg/kg/day tea extracts continuously for 9 weeks based on high-fat modeling, and the R1 + ND, P8 + ND, and P8 + HFD groups were intragastrically administered with the same amount of distilled water. During the feeding period, their body weight was measured every week. Finally, the mice were anesthetized with 0.01 g/mL pentobarbital sodium for dissection. The epididymal adipose tissues were weighed, and the cerebral cortex tissues were stored at −80 °C. The body fat ratio (%) = weight of adipose tissue/body weight × 100%.

### 2.9. Kit Detection of Lipid Levels and Oxidative Stress

Strictly followed the kit’s instructions, accurately weighed the brain tissue and cerebral cortex tissue, added 9 times the volume of homogenization medium and mechanically ground in a tissue grinder under ice-water bath conditions. After centrifugation, we took the supernatant and determined the content of TG and TC in brain tissue, SOD, CAT, GSH-Px, and MDA levels in cerebral cortex tissue.

### 2.10. Elisa Kit Detection of IL-6 and Beta-Amyloid 42 (Aβ42)

Strictly followed the ELISA kit’s instructions, accurately weighed the brain tissue andcerebral cortex tissue, added 9 times the volume of RIPA lysate and mechanically ground in a tissue grinder under ice-water bath conditions. After centrifugation (4 °C, 12,000 rpm, 20 min), the supernatant was taken, and IL-6 in brain tissue and Aβ42 in cerebral cortex tissue were determined.

### 2.11. Kit Detection of ATP

Strictly followed the kit’s instructions, accurately weighed the brain tissue, added 9 times the volume of lysate and mechanically ground in a tissue grinder under ice-water bath conditions. After centrifugation (4 °C, 12,000× *g*, 5 min), the supernatant was taken and the ATP level was determined.

### 2.12. Histological Analysis

The epididymal adipose tissues were quickly fixed in tissue fixative for more than 48 h, running overnight, dehydrated with alcohol, embedded in paraffin, and cut into 5 μm sections. The sections were deparaffinized, hydrated, stained with hematoxylin-eosin (H&E) staining, and the morphology of fat cells in the visual field was observed under a 200-fold microscope.

### 2.13. Western Blotting Analysis

The cerebral cortex tissue of mice was taken into RIPA lysate containing a protease inhibitor, and the supernatant was collected by centrifugation (4 °C, 12,000 rpm, 20 min). BCA protein analysis kit (Pierce, Grand Island, NY, USA) was used to determine the protein concentration. Equal amounts of protein for each sample were separated on 10% SDS-PAGE gel. Proteins from the gel were transferred to PVDF (Millipore, Boston, MA, USA) membrane. The membranes were blocked in 5% non-fat milk in TBS containing 0.05% Tween-20 (TBST) buffer for 1 h. We used the primary antibody diluent to dilute the primary antibody at a dilution radio of 1:1000, and incubated it overnight at 4 °C. After being washed in TBST buffer, the membranes were incubated with appropriate secondary antibodies for 90 min at room temperature. After washing with TBST, the PVDF films were reacted with the enhanced chemiluminescent substrate (Millipore, Boston, MA, USA) for 2–5 min, and the chemiluminescence gel imaging system detected the luminescence signal. Image-J software was used to analyze each band’s optical density (OD) value [42].

### 2.14. Statistical Analysis

We used GraphPad Prism 8.01 software, combined with Turkey’s multiple comparison test, a one-way ANOVA test was used to analyze the significance of difference. The results were expressed as mean ± standard deviation. The variation was judged significant by the condition of *p* < 0.05 and highly significant by *p* < 0.01. The diameter of epididymal adipocytes was statistically analyzed using Image-J software.

## 3. Results

### 3.1. Comparison of Inhibitory Activity of 30 Teas on Aging-Associated Amyloid Formation In Vitro

**Figure 1:**
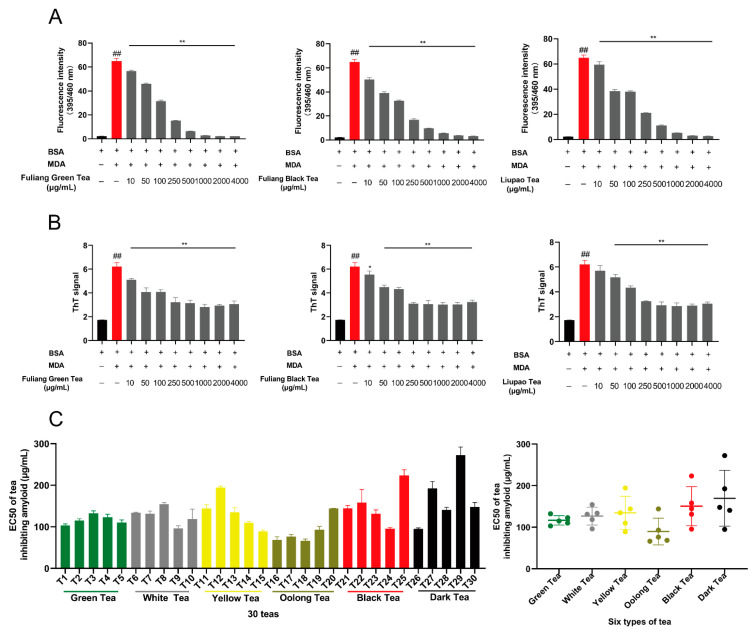
Comparison of in vitro activities of 30 teas in inhibiting the formation of aging-related amyloid. (**A**) The characteristic fluorescence of aging-related amyloid (λm/λX = 395 nm/460 nm) test results (only the results of some teas were shown); (**B**) ThT fluorescence detection of the β-sheet structure of amyloid (only showed the results of some teas); (**C**) The half effective concentration (EC50) of 30 teas to inhibit the production of aging-related amyloid. ## *p* < 0.01, compared with control group; ** *p* < 0.01, compared with BSA/MDA group, *n* = 3.

MDA and other lipid-derived aldehydes are the biomarkers of oxidative stress. They have strong electrophilic 1,3-diunsaturated aldehyde groups, which can react with biomacromolecules through the carbonyl-amino crosslinks reaction. Approximately 50% of proteins undergo carbonyl-amino crosslinks reactions in aging organisms, which promotes the formation of amyloid in the β-sheet structure [43,44]. The BSA/MDA reaction system was used to prepare aging-related amyloid. The detection results of aging-associated characteristic fluorescence (λm/λx = 395 nm/460 nm) and ThT showed that, compared with the control group (BSA), the fluorescence value of the aging-associated amyloid (BSA/MDA) group increased significantly (*p* < 0.01); compared with the modeling group, the fluorescence values of the 30 tea extracts incubation groups decreased significantly (Figure 1A,B). The results indicated that the pre-incubation of 30 tea extracts could significantly inhibit the production of amyloid (only partial results were shown). The average half-effect concentration (EC50) of the six types of tea for inhibiting the production amyloid from small to large were 89.70 ± 28.73 µg/mL (oolong tea), 116.68 ± 10.12 µg/mL (green tea), 126.88 ± 19.21 µg/mL (white tea), 134.56 ± 35.61 µg/mL (yellow tea), 150.64 ± 41.94 µg/mL (black tea), and 172.60 ± 56.60 µg/mL (dark tea) (Figure 1C). The results indicated that oolong tea had the strongest activity in avoiding the formation of aging-related amyloid in vitro, while the activities of other teas were not significantly different.

### 3.2. Correlation Analysis of Amyloid Inhibitory Activity and Catechin Content of 30 Teas

**Figure 2:**
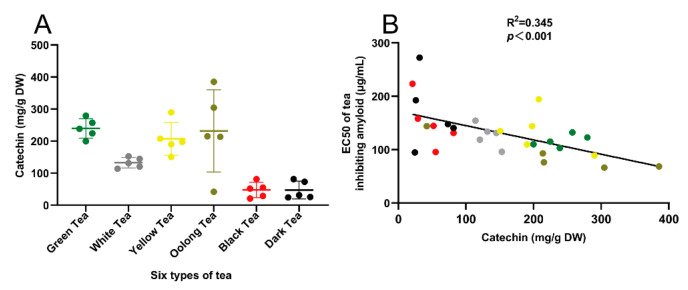
Correlation analysis between catechin content of 30 teas and the in vitro in the inhibitory activity of amyloid formation. (**A**) Comparison of catechin content in 30 teas; (**B**) Correlation between the amyloid inhibitory activity of 30 teas and catechin content. Note: the six colored dots in the picture represent six types of tea: green tea, green; white tea, grey; yellow tea, yellow; oolong tea, brown; black tea, red; dark tea, black.

The results of catechin detection showed that the catechin content of six types of tea showed a strong regularity (Figure 2A). Their dry weight (DW), from large to light, was 239.84 ± 27.22 mg/g dry weight (green tea), 232.07 ± 114.43 mg/g (oolong tea), 207.19 ± 45.64 mg/g (yellow tea), 132.68 ± 14.48 mg/g (white tea), 77.10 ± 52.35 mg/g (dark tea), and 47.65 ± 21.42 mg/g (black tea). The results showed that there was a certain correlation between the amyloid inhibitory activity of 30 teas and their respective catechin content, and the correlation coefficient was R^2^ = 0.345 (*p* < 0.001) (Figure 2B), but there was no correlation with their respective alkaloid content (see Supplementary Materials Figure S1).

### 3.3. Correlation Analysis of 30 Teas’ Antioxidant Capacity and Catechin Content

**Figure 3:**
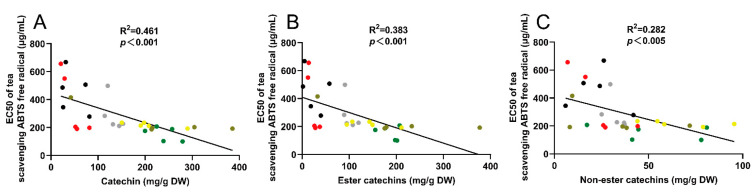
Correlation between antioxidant activity and catechin content of 30 teas. (**A**) Correlation between antioxidant activity and catechin content of 30 teas; (**B**) Correlation between antioxidant activity and ester catechins content of 30 teas; (**C**) Correlation between antioxidant activity and non-ester catechins content of 30 teas. Note: the six colored dots in the picture represent six types of tea: green tea, green; white tea, grey; yellow tea, yellow; oolong tea, brown; black tea, red; dark tea, black.

The results of correlation analysis showed that there was an excellent regularity between antioxidant activity and catechin content of 30 teas (R^2^ = 0.461, *p* < 0.001) (Figure 3A). Among them, the correlation with the content of ester catechins (R^2^ = 0.383, *p* < 0.001) (Figure 3B) was better than the correlation with the content of non-ester catechins (R^2^ = 0.282, *p* < 0.005) (Figure 3C).

### 3.4. Correlation Analysis between the Inhibition of Amyloid Formation and Antioxidant Capacity of 30 Teas

**Figure 4:**
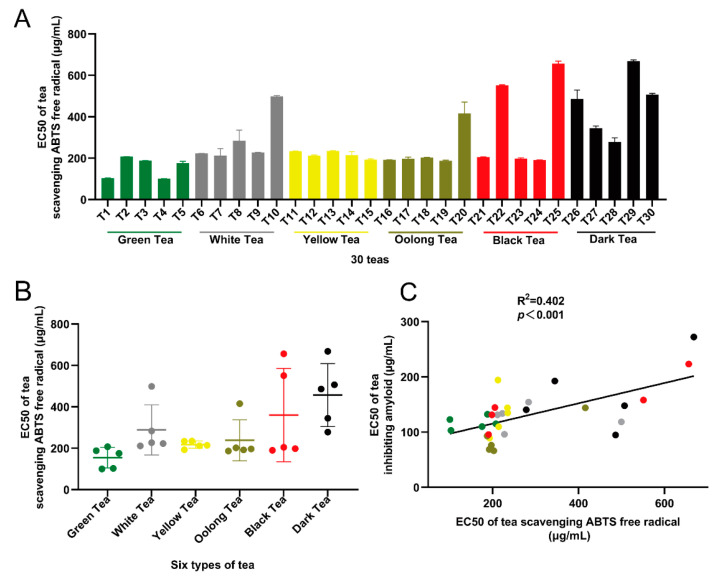
Correlation analysis between amyloid formation inhibitory activity and antioxidant activity of 30 teas. (**A**,**B**) The half effective concentration (EC50) of 30 teas to scavenge ABTS free radical; (**C**) The correlation between the in vitro amyloid formation inhibitory activity and antioxidant activity of 30 types of tea. Note: the six colored dots in the picture represent six types of tea: green tea, green; white tea, grey; yellow tea, yellow; oolong tea, brown; black tea, red; dark tea, black.

The six types of tea had great differences in the scavenging activity of ABTS free radicals. The average EC50 of the six types of tea in ascending order were:154.96 ± 44.53 µg/mL (green tea), 217.75 ± 15.81 µg/mL (yellow tea), 238.88 ± 88.53 µg/mL (oolong tea), 288.88 ± 108.14 µg/mL (white tea), 360.18 ± 201.49 µg/mL (black tea), and 456.77 ± 135.99 µg/mL (dark tea) (Figure 4B).

The result of correlation analysis was shown in Figure 4C. There was a strong positive correlation between the inhibition of amyloid formation and the antioxidant capacity (R^2^ = 0.402, *p* < 0.001). White tea (T10) and dark tea (T26) had relatively low antioxidant capacity but could significantly inhibit the formation of aging-related amyloid.

### 3.5. Improved Effect of Different Teas on Lipid Metabolism in SAMP8 Mice on High-Fat Diet

**Figure 5:**
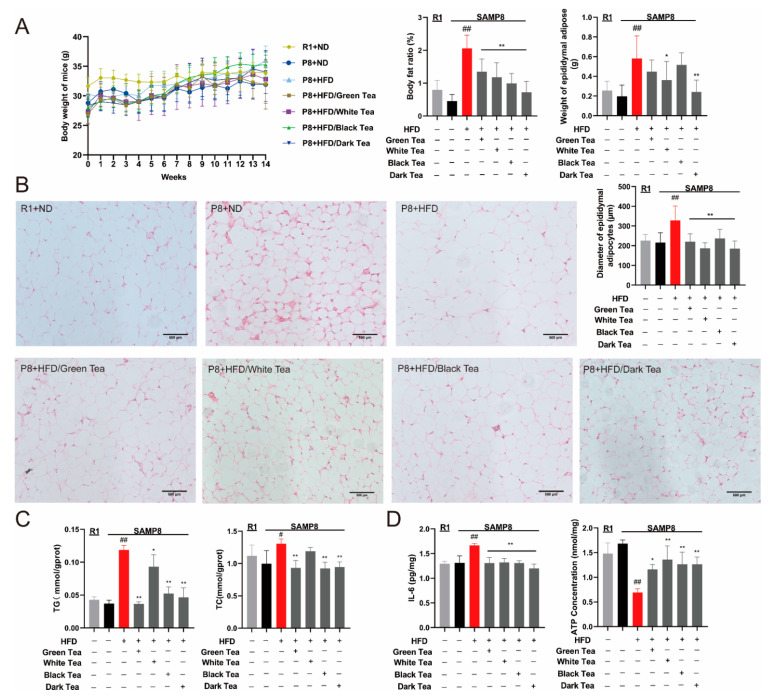
Different teas could improve lipid metabolism in SAMP8 mice on high-fat diet. (**A**) Comparison of body weight, epididymal adipose tissue weight, and body fat ratio of mice in different treatment groups; (**B**) HE staining (200×) and fat cell diameter statistics of epididymal fat tissue of mice in different treatment groups, bar = 500 µm; (**C**) Detection of lipid content in the brain tissue of mice in different treatment groups; (**D**) Detection of IL-6 and ATP content in brain tissue of mice in different treatment groups. Compared with P8 group, # *p* < 0.05, ## *p* < 0.01; Compared with HFD + P8 group, * *p* < 0.05, ** *p* < 0.01, *n* = 3. Note: green tea (T1) as control, old white tea (T9), black tea (T23) and dark tea (T26).

In order to compare the in vivo activity of different teas, a mouse model of premature aging SAMP8 high-fat diet was established, and different tea extracts were administered to the stomach. The results showed that compared with normal diet groups (R1 + ND and P8 + ND), the premature aging SAMP8 high-fat model group (P8 + HFD) had significantly increased epididymal adipose tissue weight and body fat ratio (*p* < 0.01). Compared with the P8 + HFD group, epididymal fat weight and body fat ratio decreased in different tea treatment groups, and the lowest was the dark tea group (P8 + HFD/Dark tea (T26)) (*p* < 0.01) (Figure 5A). The result of HE staining showed that the diameter of epididymal adipocytes in the P8 + HFD group was larger than that in R1 + ND and P8 + ND groups (*p* < 0.01). Compared with the P8 + HFD group, the epididymal adipocytes diameter in the different tea treatment groups was decreased (*p* < 0.01) (Figure 5B).

TG and TC kits were used to detect lipid level in brain tissue. The results showed that compared with the R1 + ND and P8 + ND groups, the levels of TG and TC in brain tissue of P8 + HFD group were increased significantly (*p* < 0.01 or *p* < 0.05). Compared with the P8 + HFD group, TG and TC levels in brain tissue were decreased in all tea-treated groups (Figure 5C). The results of IL-6 and ATP showed that compared with the R1 + ND and P8 + ND groups, the IL-6 content was significantly increased (*p* < 0.01), the ATP level was significantly decreased (*p* < 0.01); Compared with the P8 + HFD group, the IL-6 content of the different tea treatment groups were decreased significantly (*p* < 0.05), and the ATP level was increased (*p* < 0.05) (Figure 5D). The results indicate that different teas have a regulatory effect on age-related lipid metabolism disorders. Among them, old white tea (T9), black tea (T23), and dark tea (T26) are more active than green tea (T1).

### 3.6. Inhibitory Effects of Different Teas on the Formation of Amyloid in the Cerebral Cortex of SAMP8 Mice on High-Fat Diet

**Figure 6:**
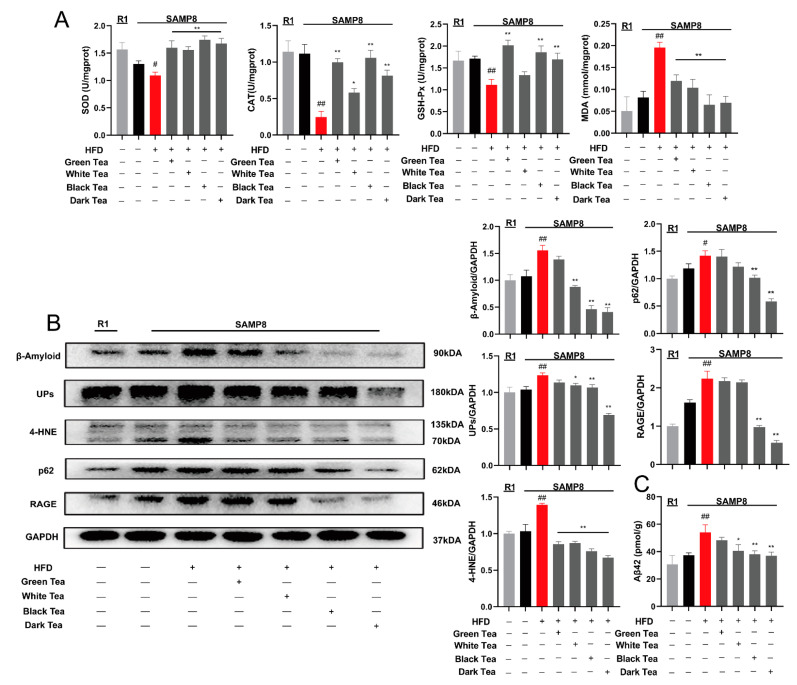
Comparison of redox level and β-amyloid protein formation in the cerebral cortex of mice in different tea treatment groups. (**A**) Detection of oxidative stress index; (**B**) Western blotting of the cerebral cortex; (**C**) Content measurement of Aβ42 of the cerebral cortex based on ELISA. Compared with P8 group, # *p* < 0.05, ## *p* < 0.01; Compared with HFD + P8 group, * *p* < 0.05, ** *p* < 0.01, *n* = 3. Note: green tea (T1), aged white tea (T9), black tea (T23) and dark tea (T26).

The test results of kit showed that compared with R1 + ND and P8 + ND groups, the SOD, CAT, and GSH-Px activities in the cerebral cortex of the P8 + HFD group decreased, and the MDA contents were increased. Among them, CAT and MDA had significant differences (*p* < 0.01). Compared with the P8 + HFD group, the antioxidant kinases activities of the different tea treatment groups increased and the MDA content was significantly decreased (*p* < 0.01) (Figure 6A). Western blotting results showed that compared with the R1 + ND and P8 + ND groups, the levels of β-amyloid, 4-HNE, UPs, p62, and RAGE in the P8 + HFD group were significantly increased (*p* < 0.01 or *p* < 0.05). Compared with the P8 + HFD group, the levels of β-amyloid, 4-HNE, UPs, p62, and RAGE in the different tea treated groups were significantly reduced (*p* < 0.01) (Figure 6B). The results indicate that black tea (T23), aged white tea (T9) and dark tea (T26) have a significant inhibitory effect on the formation of β-amyloid mediated by age-related lipid metabolism disorders, and their efficacy is better than green tea. The ELISA result showed that, compared with the R1 + ND and P8 + ND groups, the content of Aβ42 in the P8 + HFD cerebral cortex was increased (*p* < 0.01). Compared with P8 + HFD mice, different tea treatment groups reduced the content of Aβ42, especially the black tea and dark tea treatment groups had significant reduction effects (*p* < 0.01) (Figure 6C).

## 4. Discussion

The results of this study indicated that six types of tea had a significant in vitro inhibitory effect on the aging-related amyloid formation activity, and the inhibitory activity was positively correlated with the catechins content and antioxidant capacity (Figure 1, Figure 2, Figure 3 and Figure 4). Based on in vitro experiments, green tea (T1), aged white tea (T9), black tea (T23), and dark tea (T26) were selected to further verify the in vivo activity of teas with different fermentation levels to inhibit amyloid formation. The results indicated that in the premature aging SAMP8 high-fat model mice, aged white tea (T9), black tea (T23), and black tea (T26) had the activity of improving lipid metabolism and inhibiting amyloid production, and their efficacy was better than that of green tea (T1) (Figure 5 and Figure 6).

Aging-related amyloid has highly active structures such as conjugation and electron-deficient, and can be continuously cross-linked with nucleophilic amino acid residues such as Cys, His, and Lys of biological macromolecules [20]. Aging-related amyloid can destroy the cell structure and function of the lysosome, mitochondria, cell membrane, etc., and further promote the formation and accumulation of amyloid toxic β-sheet structure [22]. In our study, 30 teas significantly inhibit aging-related amyloid formation by avoiding carbonyl-amino crosslinks and amyloid toxic β-sheet structure formation (Figure 1 and Figure 2).

Application of an antioxidant or inhibition of lipid peroxidation products MDA, 4-HNE, and other carbonyl toxic compounds are effective methods to inhibit the formation of aging-related amyloid. Tea polyphenols, especially EGCG, have significant antioxidant and carbonyl capture activities [45]. Catechins account for approximately 50–80% of tea polyphenols, and they all have 2-phenylbenzopyran as the main structure. The structure–activity analysis shows that the resorcinol structure of the A ring can generate high nucleophilic centers at C6 and C8, and the ortho-dihydroxy or trihydroxy group of B ring is a strong electron donor, which are the antioxidant and reducing centers of catechins [46,47]. The results of LC-MS and NMR indicate that unsaturated carbonyl compounds such as MDA mainly reacted with 6 and 8 positions of the catechin A ring [48,49,50,51,52]. Compared with black tea and dark tea, green tea, white tea, yellow tea, and oolong tea are rich in catechins, which can inhibit the formation of aging-related amyloid through the following four mechanisms: (1) antioxidation; (2) by capturing toxic carbonyl compounds such as MDA and 4-HNE; (3) inhibiting the aggregation of amyloid-β (Aβ) by inhibiting the production of β-sheet structure [32,53], or (4) by binding to electron-rich groups such as lysine, it competitively inhibits the binding of carbonyl compounds to proteins [54]. In our study, 30 teas significantly inhibit aging-related amyloid formation by avoiding carbonyl-amino crosslinks and amyloid toxic β-sheet structure formation (Figure 1 and Figure 2). Our study indicated that oolong tea had the strongest activity in preventing aging-related amyloid formation in vitro, while the other five types of tea had no significant difference in activity (Figure 1C). The amyloid inhibitory activity had a certain correlation with the catechin content of each tea in 30 teas (Figure 3).

The research data of green tea and its core functional component EGCG are relatively systematic, and research on fully fermented teas, such as black tea and dark tea, is rare. Literature data gradually show that the catechin content and antioxidant capacity of fermented teas such as black tea and dark tea are not high in vitro, but they have better antioxidant and metabolic functions in the body [55,56,57]. Aging and high-fat diet are the main factors leading to amyloid deposition [58,59,60]. Black tea and its core functional components, theaflavins, have a significant inhibitory effect on the formation of amyloid [61,62]. Black tea is fully fermented tea. In processing, catechins undergo oxidative polymerization to form a series of oxidized polyphenols such as theaflavins, thearubigins, and theabrownines [63,64]. Like EGCG, theaflavins can stimulate Aβ and α nuclide (αS) to assemble non-toxic spherical polymers, and are effective inhibitors of Aβ and αS fiber formation. Mechanism studies have shown that theaflavins have a stronger conjugation effect and phenol-qhinone balance structure than catechins, leading to stronger proton supply and nucleophilicity, which is essential to inhibit the aggregation of Aβ [65,66,67,68,69,70].

White tea belongs to light-fermented tea, and dark tea belongs to post-fermented tea. The longer the two types of tea are stored, the better the efficacy and flavor quality [71,72]. During the storage process, the components of tea are transformed, and the active components such as tea pigment, organic acid, and tea polysaccharide are gradually increased, and the quality and efficacy of tea are gradually improved [73]. The results indicate that dark tea has significant antioxidant activity in vivo, and the antioxidant activity of Liupao dark tea is even better than that of some green tea [74]. These results are consistent with our results. The latest research shows that catechins and theanine gradually condense during the fermentation process of white tea and dark tea to form a kind of flavoalkaloids products condensed at C8 position. Efficacy and mechanism evaluations show that the C8 condensed flavoalkaloids have significant antioxidant and inhibitory activities on the formation of amyloid in premature aging SAMP8 mice [34]. The results show that aged white tea, black tea, and dark tea have lower catechin content than green tea, but still have significant inhibitory activity on amyloid formation. Our study results indicated that different tea types have a significant inhibitory effect on the formation of β-amyloid mediated by age-related lipid metabolism disorders, and the in vivo activity of fully fermented teas was better than that of green tea. The action mechanism was related to antioxidation, anti-inflammatory, and improving lipid metabolism (Figure 5 and Figure 6).

## 5. Conclusions

In conclusion, the six types of Chinese tea have significant inhibitory activity on aging-related amyloid formation. Thirty teas had significant inhibitory effects on the formation of aging-related amyloid in vitro, and the mechanism of action was positively correlated with the content of catechins and antioxidant capacity. The catechins content and antioxidant capacity of aged white tea, black tea, and dark tea were not high, but they could significantly inhibit the formation of amyloid mediated by aging and high-fat diet, and the effects were even better than that of green tea. Aged tea, such as old white tea and dark tea, will increase the flavor quality and efficacy as the storage time increases. More and more studies have shown that aged dark tea has significant antioxidant, metabolic regulation and amyloid inhibitory activity in the body. However, the influence of the storage process on the quality and activity of fermented teas is complex and multifaceted, and research in this tea is still lacking. Therefore, this field requires systematic research and safety evaluation to provide a theoretical basis for the in-depth research and application of fermented tea and post-fermented tea to prevent and treat age-related metabolic disorders and degenerative diseases.

## Figures and Tables

**Table 1:** The information of 30 Chinese teas.

No.	Name	Storage Years	Category	Production Place
T1	Fuliang Green Tea	0	Green Tea (Unfermented)	Jiangxi
T2	Guzhang Maojian	0		Hunan
T3	Huangjin Tea	0		Hunan
T4	Enshi Green Tea	0		Hubei
T5	Fengyu	0		Sichuan
T6	Baihao Yinzhen	4	White Tea (Slight-fermented)	Fujian
T7	Huangye Yinzhen	2		Fujian
T8	Gongmei	5		Fujian
T9	Baimudan	6		Fujian
T10	Shoumei	0		Fujian
T11	Junshan Yinzhen	6	Yellow Tea (Light-fermented)	Hunan
T12	Mengding Huangya	1		Sichuan
T13	Bianhuang	0		Hunan
T14	Huangdacha	0		Anhui
T15	Maojian	0		Hunan
T16	Hongyin	6	Oolong Tea (Semi-fermented)	Guangdong
T17	Xuepian	6		Guangdong
T18	Baiye	6		Guangdong
T19	Dancong	4		Guangdong
T20	Dahongpao	4		Fujian
T21	Yunnan Jinzhen	6	Black Tea (Fully-fermented)	Yunnan
T22	Dianhong	1		Yunnan
T23	Fuliang Black Tea	0		Jiangxi
T24	Yingde Black Tea	0		Guangdong
T25	Qihong	0		Anhui
T26	Liupao Tea	7	Dark Tea (Post-fermented)	Guangxi
T27	Yangloudong	8		Hubei
T28	Jingwei Fuzhuan	6		Shanxi
T29	Pu-erh	6		Yunnan
T30	Jiangkang Fuzhuan	6		Hunan

## Data Availability

Data is contained within the article and Supplementary Material.

## Supplementary Materials

The following are available online at https://www.mdpi.com/article/10.3390/antiox10101513/s1, Figure S1: Correlation analysis between alkaloid and theabrownines contents of different teas and inhibition of amyloid formation in vitro.
