# Supplementary material for: Inhibitory Effects of Six Types of Tea on Aging and High-Fat Diet-Related Amyloid Formation Activities
Source: Antioxidants (Basel). 2021 Sep 24;10(10):1513. doi: 10.3390/antiox10101513 (PMC8533055; doi:10.3390/antiox10101513)
Supplement: Supplementary file 1 [file antioxidants-10-01513-s001.zip › antioxidants-1317562-SI.pdf]

Inhibitory Effects of Six Types of Tea on Aging and High-Fat Diet-Related Amyloid Formation Activities

Juan Wan [1,2,3], Meiyan Feng [1,2,3], Wenjing Pan [1,2,3], Xin Zheng [1,2,3], Xinya Xie [1,2,3], Baozhu Hu [1,2,3], Cuiqin Teng [4],
Yingzi Wang [1,2,3], Zhonghua Liu [1,2,3,*], Jianhua Wu [4] and Shuxian Cai [1,2,3,*]

[1] National Research Center of Engineering Technology for Utilization of Botanical Functional Ingredients, Hunan Agricultural University, Changsha 410128, China; wanjuan@stu.hunau.edu.cn (J.W.);
fengmeiyan@stu.hunau.edu.cn (M.F.); pwj0204@stu.hunau.edu.cn (W.P.);
zhengxin@stu.hunau.edu.cn (X.Z.); xxy999@stu.hunau.edu.cn (X.X.);
HU0707@stu.hunau.edu.cn (B.H.); wyz19860908@163.com (Y.W.)
[2] Key Laboratory of Ministry of Education for Tea Science, Hunan Agricultural University,
Changsha 410128, China
[3] Co-Innovation Center of Education Ministry for Utilization of Botanical Functional Ingredients, Hunan Agricultural University, Changsha 410128, China
[4] Wuzhou Institute of Agricultural, Wuzhou 543003, China; teng3000@163.com (C.T.); wjh0056@163.com (J.W.)
* Correspondence: caishuxian@hunau.edu.cn (S.C.); zhonghua-liu@hunau.edu.cn (Z.L.);
Tel.: +86-138-7313-1191 (S.C.); +86-138-0749-2258 (Z.L.)

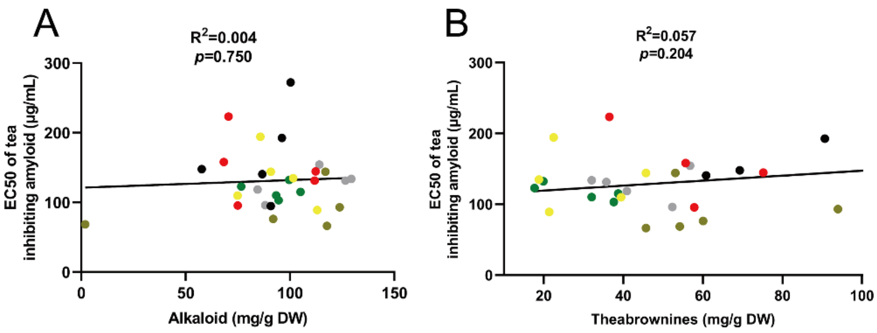

Figure S1. Correlation analysis between alkaloid and theabrownines contents of different teas and inhibition of amyloid formation in vitro.
